# Using the framework method for the analysis of qualitative data in multi-disciplinary health research

**DOI:** 10.1186/1471-2288-13-117

**Published:** 2013-09-18

**Authors:** Nicola K Gale, Gemma Heath, Elaine Cameron, Sabina Rashid, Sabi Redwood

**Affiliations:** 1 Health Services Management Centre, University of Birmingham, Park House, 40 Edgbaston Park Road, Birmingham B15 2RT, UK; 2 School of Health and Population Sciences, University of Birmingham, Edgbaston, Birmingham B15 2TT, UK; 3 School of Life and Health Sciences, Aston University, Aston Triangle, Birmingham B4 7ET, UK; 4 East and North Hertfordshire NHS Trust, Lister hospital, Coreys Mill Lane, Stevenage SG1 4AB, UK

**Keywords:** Qualitative research, Qualitative content analysis, Multi-disciplinary research

## Abstract

**Background:**

The Framework Method is becoming an increasingly popular approach to the management and analysis of qualitative data in health research. However, there is confusion about its potential application and limitations.

**Discussion:**

The article discusses when it is appropriate to adopt the Framework Method and explains the procedure for using it in multi-disciplinary health research teams, or those that involve clinicians, patients and lay people. The stages of the method are illustrated using examples from a published study.

**Summary:**

Used effectively, with the leadership of an experienced qualitative researcher, the Framework Method is a systematic and flexible approach to analysing qualitative data and is appropriate for use in research teams even where not all members have previous experience of conducting qualitative research.

## 

The Framework Method for the management and analysis of qualitative data has been used since the 1980s [1]. The method originated in large-scale social policy research but is becoming an increasingly popular approach in medical and health research; however, there is some confusion about its potential application and limitations. In this article we discuss when it is appropriate to use the Framework Method and how it compares to other qualitative analysis methods. In particular, we explore how it can be used in multi-disciplinary health research teams. Multi-disciplinary and mixed methods studies are becoming increasingly commonplace in applied health research. As well as disciplines familiar with qualitative research, such as nursing, psychology and sociology, teams often include epidemiologists, health economists, management scientists and others. Furthermore, applied health research often has clinical representation and, increasingly, patient and public involvement [2]. We argue that while leadership is undoubtedly required from an experienced qualitative methodologist, non-specialists from the wider team can and should be involved in the analysis process. We then present a step-by-step guide to the application of the Framework Method, illustrated using a worked example (See Additional File 1) from a published study [3] to illustrate the main stages of the process. Technical terms are included in the glossary (below). Finally, we discuss the strengths and limitations of the approach.

## Glossary of key terms used in the Framework Method

Analytical framework: A set of codes organised into categories that have been jointly developed by researchers involved in analysis that can be used to manage and organise the data. The framework creates a new structure for the data (rather than the full original accounts given by participants) that is helpful to summarize/reduce the data in a way that can support answering the research questions.

Analytic memo: A written investigation of a particular concept, theme or problem, reflecting on emerging issues in the data that captures the analytic process (see Additional file 1, Section 7).

Categories: During the analysis process, codes are grouped into clusters around similar and interrelated ideas or concepts. Categories and codes are usually arranged in a tree diagram structure in the analytical framework. While categories are closely and explicitly linked to the raw data, developing categories is a way to start the process of abstraction of the data (i.e. towards the general rather than the specific or anecdotal).

Charting: Entering summarized data into the Framework Method matrix (see Additional File 1, Section 6).

Code: A descriptive or conceptual label that is assigned to excerpts of raw data in a process called ‘coding’ (see Additional File 1, Section 3).

Data: Qualitative data usually needs to be in textual form before analysis. These texts can either be elicited texts (written specifically for the research, such as food diaries), or extant texts (pre-existing texts, such as meeting minutes, policy documents or weblogs), or can be produced by transcribing interview or focus group data, or creating ‘field’ notes while conducting participant-observation or observing objects or social situations.

Indexing: The systematic application of codes from the agreed analytical framework to the whole dataset (see Additional File 1, Section 5).

Matrix: A spreadsheet contains numerous cells into which summarized data are entered by codes (columns) and cases (rows) (see Additional File 1, Section 6).

Themes: Interpretive concepts or propositions that describe or explain aspects of the data, which are the final output of the analysis of the whole dataset. Themes are articulated and developed by interrogating data categories through comparison between and within cases. Usually a number of categories would fall under each theme or sub-theme [3].

Transcript: A written verbatim (word-for-word) account of a verbal interaction, such as an interview or conversation.

## Background

The Framework Method sits within a broad family of analysis methods often termed thematic analysis or qualitative content analysis. These approaches identify commonalities and differences in qualitative data, before focusing on relationships between different parts of the data, thereby seeking to draw descriptive and/or explanatory conclusions clustered around themes. The Framework Method was developed by researchers, Jane Ritchie and Liz Spencer, from the Qualitative Research Unit at the National Centre for Social Research in the United Kingdom in the late 1980s for use in large-scale policy research [1]. It is now used widely in other areas, including health research [3-12]. Its defining feature is the matrix output: rows (cases), columns (codes) and ‘cells’ of summarised data, providing a structure into which the researcher can systematically reduce the data, in order to analyse it by case and by code [1]. Most often a ‘case’ is an individual interviewee, but this can be adapted to other units of analysis, such as predefined groups or organisations. While in-depth analyses of key themes can take place across the whole data set, the views of each research participant remain connected to other aspects of their account within the matrix so that the context of the individual’s views is not lost. Comparing and contrasting data is vital to qualitative analysis and the ability to compare with ease data *across* cases as well as *within* individual cases is built into the structure and process of the Framework Method.

The Framework Method provides clear steps to follow and produces highly structured outputs of summarised data. It is therefore useful where multiple researchers are working on a project, particularly in multi-disciplinary research teams were not all members have experience of qualitative data analysis, and for managing large data sets where obtaining a holistic, descriptive overview of the entire data set is desirable. However, caution is recommended before selecting the method as it is not a suitable tool for analysing all types of qualitative data or for answering all qualitative research questions, nor is it an ‘easy’ version of qualitative research for quantitative researchers. Importantly, the Framework Method cannot accommodate highly heterogeneous data, i.e. data must cover similar topics or key issues so that it is possible to categorize it. Individual interviewees may, of course, have very different views or experiences in relation to each topic, which can then be compared and contrasted. The Framework Method is most commonly used for the thematic analysis of semi-structured interview transcripts, which is what we focus on in this article, although it could, in principle, be adapted for other types of textual data [13], including documents, such as meeting minutes or diaries [12], or field notes from observations [10].

For quantitative researchers working with qualitative colleagues or when exploring qualitative research for the first time, the nature of the Framework Method is seductive because its methodical processes and ‘spreadsheet’ approach seem more closely aligned to the quantitative paradigm [14]. Although the Framework Method is a highly systematic method of categorizing and organizing what may seem like unwieldy qualitative data, it is not a panacea for problematic issues commonly associated with qualitative data analysis such as how to make analytic choices and make interpretive strategies visible and auditable. Qualitative research skills are required to appropriately interpret the matrix, and facilitate the generation of descriptions, categories, explanations and typologies. Moreover, reflexivity, rigour and quality are issues that are requisite in the Framework Method just as they are in other qualitative methods. It is therefore essential that studies using the Framework Method for analysis are overseen by an experienced qualitative researcher, though this does not preclude those new to qualitative research from contributing to the analysis as part of a wider research team.

There are a number of approaches to qualitative data analysis, including those that pay close attention to language and how it is being used in social interaction such as discourse analysis [15] and ethnomethodology [16]; those that are concerned with experience, meaning and language such as phenomenology [17,18] and narrative methods [19]; and those that seek to develop theory derived from data through a set of procedures and interconnected stages such as Grounded Theory [20,21]. Many of these approaches are associated with specific disciplines and are underpinned by philosophical ideas which shape the process of analysis [22]. The Framework Method, however, is not aligned with a particular epistemological, philosophical, or theoretical approach. Rather it is a flexible tool that can be adapted for use with many qualitative approaches that aim to generate themes.

The development of themes is a common feature of qualitative data analysis, involving the systematic search for patterns to generate full descriptions capable of shedding light on the phenomenon under investigation. In particular, many qualitative approaches use the ‘constant comparative method’ , developed as part of Grounded Theory, which involves making systematic comparisons across cases to refine each theme [21,23]. Unlike Grounded Theory, the Framework Method is not necessarily concerned with generating social theory, but can greatly facilitate constant comparative techniques through the review of data across the matrix.

Perhaps because the Framework Method is so obviously systematic, it has often, as other commentators have noted, been conflated with a deductive approach to qualitative analysis [13,14]. However, the tool itself has no allegiance to either inductive or deductive thematic analysis; where the research sits along this inductive-deductive continuum depends on the research question. A question such as, ‘Can patients give an accurate biomedical account of the onset of their cardiovascular disease?’ is essentially a yes/no question (although it may be nuanced by the extent of their account or by appropriate use of terminology) and so requires a deductive approach to both data collection and analysis (e.g. structured or semi-structured interviews and directed qualitative content analysis [24]). Similarly, a deductive approach may be taken if basing analysis on a pre-existing theory, such as behaviour change theories, for example in the case of a research question such as ‘How does the Theory of Planned Behaviour help explain GP prescribing?’ [11]. However, a research question such as, ‘How do people construct accounts of the onset of their cardiovascular disease?’ would require a more inductive approach that allows for the unexpected, and permits more socially-located responses [25] from interviewees that may include matters of cultural beliefs, habits of food preparation, concepts of ‘fate’, or links to other important events in their lives, such as grief, which cannot be predicted by the researcher in advance (e.g. an interviewee-led open ended interview and grounded theory [20]). In all these cases, it may be appropriate to use the Framework Method to manage the data. The difference would become apparent in how themes are selected: in the deductive approach, themes and codes are pre-selected based on previous literature, previous theories or the specifics of the research question; whereas in the inductive approach, themes are generated from the data though open (unrestricted) coding, followed by refinement of themes. In many cases, a combined approach is appropriate when the project has some specific issues to explore, but also aims to leave space to discover other unexpected aspects of the participants’ experience or the way they assign meaning to phenomena. In sum, the Framework Method can be adapted for use with deductive, inductive, or combined types of qualitative analysis. However, there are some research questions where analysing data by case and theme is not appropriate and so the Framework Method should be avoided. For instance, depending on the research question, life history data might be better analysed using narrative analysis [19]; recorded consultations between patients and their healthcare practitioners using conversation analysis [26]; and documentary data, such as resources for pregnant women, using discourse analysis [27].

It is not within the scope of this paper to consider study design or data collection in any depth, but before moving on to describe the Framework Method analysis process, it is worth taking a step back to consider briefly what needs to happen before analysis begins. The selection of analysis method should have been considered at the proposal stage of the research and should fit with the research questions and overall aims of the study. Many qualitative studies, particularly ones using inductive analysis, are emergent in nature; this can be a challenge and the researchers can only provide an “imaginative rehearsal” of what is to come [28]. In mixed methods studies, the role of the qualitative component within the wider goals of the project must also be considered. In the data collection stage, resources must be allocated for properly trained researchers to conduct the qualitative interviewing because it is a highly skilled activity. In some cases, a research team may decide that they would like to use lay people, patients or peers to do the interviews [29-32] and in this case they must be properly trained and mentored which requires time and resources. At this early stage it is also useful to consider whether the team will use Computer Assisted Qualitative Data Analysis Software (CAQDAS), which can assist with data management and analysis.

As any form of qualitative or quantitative analysis is not a purely technical process, but influenced by the characteristics of the researchers and their disciplinary paradigms, critical reflection throughout the research process is paramount, including in the design of the study, the construction or collection of data, and the analysis. All members of the team should keep a research diary, where they record reflexive notes, impressions of the data and thoughts about analysis throughout the process. Experienced qualitative researchers become more skilled at sifting through data and analysing it in a rigorous and reflexive way. They cannot be too attached to certainty, but must remain flexible and adaptive throughout the research in order to generate rich and nuanced findings that embrace and explain the complexity of real social life and can be applied to complex social issues. It is important to remember when using the Framework Method that, unlike quantitative research where data collection and data analysis are strictly sequential and mutually exclusive stages of the research process, in qualitative analysis there is, to a greater or lesser extent depending on the project, ongoing interplay between data collection, analysis, and theory development. For example, new ideas or insights from participants may suggest potentially fruitful lines of enquiry, or close analysis might reveal subtle inconsistencies in an account which require further exploration.

### Procedure for analysis

#### Stage 1: Transcription

A good quality audio recording and, ideally, a *verbatim* (word for word) transcription of the interview is needed. For Framework Method analysis, it is not necessarily important to include the conventions of dialogue transcriptions which can be difficult to read (e.g. pauses or two people talking simultaneously), because the content is what is of primary interest. Transcripts should have large margins and adequate line spacing for later coding and making notes. The process of transcription is a good opportunity to become immersed in the data and is to be strongly encouraged for new researchers. However, in some projects, the decision may be made that it is a better use of resources to outsource this task to a professional transcriber.

#### Stage 2: Familiarisation with the interview

Becoming familiar with the whole interview using the audio recording and/or transcript and any contextual or reflective notes that were recorded by the interviewer is a vital stage in interpretation. It can also be helpful to re-listen to all or parts of the audio recording. In multi-disciplinary or large research projects, those involved in analysing the data may be different from those who conducted or transcribed the interviews, which makes this stage particularly important. One margin can be used to record any analytical notes, thoughts or impressions.

#### Stage 3: Coding

After familiarization, the researcher carefully reads the transcript line by line, applying a paraphrase or label (a ‘code’) that describes what they have interpreted in the passage as important. In more inductive studies, at this stage ‘open coding’ takes place, i.e. coding anything that might be relevant from as many different perspectives as possible. Codes could refer to substantive things (e.g. particular behaviours, incidents or structures), values (e.g. those that inform or underpin certain statements, such as a belief in evidence-based medicine or in patient choice), emotions (e.g. sorrow, frustration, love) and more impressionistic/methodological elements (e.g. interviewee found something difficult to explain, interviewee became emotional, interviewer felt uncomfortable) [33]. In purely deductive studies, the codes may have been pre-defined (e.g. by an existing theory, or specific areas of interest to the project) so this stage may not be strictly necessary and you could just move straight onto indexing, although it is generally helpful even if you are taking a broadly deductive approach to do some open coding on at least a few of the transcripts to ensure important aspects of the data are not missed. Coding aims to classify all of the data so that it can be compared systematically with other parts of the data set. At least two researchers (or at least one from each discipline or speciality in a multi-disciplinary research team) should independently code the first few transcripts, if feasible. Patients, public involvement representatives or clinicians can also be productively involved at this stage, because they can offer alternative viewpoints thus ensuring that one particular perspective does not dominate. It is vital in inductive coding to look out for the unexpected and not to just code in a literal, descriptive way so the involvement of people from different perspectives can aid greatly in this. As well as getting a holistic impression of what was said, coding line-by-line can often alert the researcher to consider that which may ordinarily remain invisible because it is not clearly expressed or does not ‘fit’ with the rest of the account. In this way the developing analysis is challenged; to reconcile and explain anomalies in the data can make the analysis stronger. Coding can also be done digitally using CAQDAS, which is a useful way to keep track automatically of new codes. However, some researchers prefer to do the early stages of coding with a paper and pen, and only start to use CAQDAS once they reach Stage 5 (see below).

#### Stage 4: Developing a working analytical framework

After coding the first few transcripts, all researchers involved should meet to compare the labels they have applied and agree on a set of codes to apply to all subsequent transcripts. Codes can be grouped together into categories (using a tree diagram if helpful), which are then clearly defined. This forms a working analytical framework. It is likely that several iterations of the analytical framework will be required before no additional codes emerge. It is always worth having an ‘other’ code under each category to avoid ignoring data that does not fit; the analytical framework is never ‘final’ until the last transcript has been coded.

#### Stage 5: Applying the analytical framework

The working analytical framework is then applied by indexing subsequent transcripts using the existing categories and codes. Each code is usually assigned a number or abbreviation for easy identification (and so the full names of the codes do not have to be written out each time) and written directly onto the transcripts. Computer Assisted Qualitative Data Analysis Software (CAQDAS) is particularly useful at this stage because it can speed up the process and ensures that, at later stages, data is easily retrievable. It is worth noting that unlike software for statistical analyses, which actually carries out the calculations with the correct instruction, putting the data into a qualitative analysis software package does not analyse the data; it is simply an effective way of storing and organising the data so that they are accessible for the analysis process.

#### Stage 6: Charting data into the framework matrix

Qualitative data are voluminous (an hour of interview can generate 15–30 pages of text) and being able to manage and summarize (reduce) data is a vital aspect of the analysis process. A spreadsheet is used to generate a matrix and the data are ‘charted’ into the matrix. Charting involves summarizing the data by category from each transcript. Good charting requires an ability to strike a balance between reducing the data on the one hand and retaining the original meanings and ‘feel’ of the interviewees’ words on the other. The chart should include references to interesting or illustrative quotations. These can be tagged automatically if you are using CAQDAS to manage your data (N-Vivo version 9 onwards has the capability to generate framework matrices), or otherwise a capital ‘Q’, an (anonymized) transcript number, page and line reference will suffice. It is helpful in multi-disciplinary teams to compare and contrast styles of summarizing in the early stages of the analysis process to ensure consistency within the team. Any abbreviations used should be agreed by the team. Once members of the team are familiar with the analytical framework and well practised at coding and charting, on average, it will take about half a day per hour-long transcript to reach this stage. In the early stages, it takes much longer.

#### Stage 7: Interpreting the data

It is useful throughout the research to have a separate note book or computer file to note down impressions, ideas and early interpretations of the data. It may be worth breaking off at any stage to explore an interesting idea, concept or potential theme by writing an analytic memo [20,21] to then discuss with other members of the research team, including lay and clinical members. Gradually, characteristics of and differences between the data are identified, perhaps generating typologies, interrogating theoretical concepts (either prior concepts or ones emerging from the data) or mapping connections between categories to explore relationships and/or causality. If the data are rich enough, the findings generated through this process can go beyond description of particular cases to explanation of, for example, reasons for the emergence of a phenomena, predicting how an organisation or other social actor is likely to instigate or respond to a situation, or identifying areas that are not functioning well within an organisation or system. It is worth noting that this stage often takes longer than anticipated and that any project plan should ensure that sufficient time is allocated to meetings and individual researcher time to conduct interpretation and writing up of findings (see Additional file 1, Section 7).

## Discussion

The Framework Method has been developed and used successfully in research for over 25 years, and has recently become a popular analysis method in qualitative health research. The issue of how to assess quality in qualitative research has been highly debated [20,34-40], but ensuring rigour and transparency in analysis is a vital component. There are, of course, many ways to do this but in the Framework Method the following are helpful:

•Summarizing the data during charting, as well as being a practical way to reduce the data, means that all members of a multi-disciplinary team, including lay, clinical and (quantitative) academic members can engage with the data and offer their perspectives during the analysis process without necessarily needing to read all the transcripts or be involved in the more technical parts of analysis.

•Charting also ensures that researchers pay close attention to describing the data using each participant’s own subjective frames and expressions in the first instance, before moving onto interpretation.

•The summarized data is kept within the wider context of each case, thereby encouraging thick description that pays attention to complex layers of meaning and understanding [38].

•The matrix structure is visually straightforward and can facilitate recognition of patterns in the data by any member of the research team, including through drawing attention to contradictory data, deviant cases or empty cells.

•The systematic procedure (described in this article) makes it easy to follow, even for multi-disciplinary teams and/or with large data sets.

•It is flexible enough that non-interview data (such as field notes taken during the interview or reflexive considerations) can be included in the matrix.

•It is not aligned with a particular epistemological viewpoint or theoretical approach and therefore can be adapted for use in inductive or deductive analysis or a combination of the two (e.g. using pre-existing theoretical constructs deductively, then revising the theory with inductive aspects; or using an inductive approach to identify themes in the data, before returning to the literature and using theories deductively to help further explain certain themes).

•It is easy to identify relevant data extracts to illustrate themes and to check whether there is sufficient evidence for a proposed theme.

•Finally, there is a clear audit trail from original raw data to final themes, including the illustrative quotes.

There are also a number of potential pitfalls to this approach:

•The systematic approach and matrix format, as we noted in the background, is intuitively appealing to those trained quantitatively but the ‘spreadsheet’ look perhaps further increases the temptation for those without an in-depth understanding of qualitative research to attempt to quantify qualitative data (e.g. “13 out of 20 participants said X). This kind of statement is clearly meaningless because the sampling in qualitative research is not designed to be representative of a wider population, but purposive to capture diversity around a phenomenon [41].

•Like all qualitative analysis methods, the Framework Method is time consuming and resource-intensive. When involving multiple stakeholders and disciplines in the analysis and interpretation of the data, as is good practice in applied health research, the time needed is extended. This time needs to be factored into the project proposal at the pre-funding stage.

•There is a high training component to successfully using the method in a new multi-disciplinary team. Depending on their role in the analysis, members of the research team may have to learn how to code, index, and chart data, to think reflexively about how their identities and experience affect the analysis process, and/or they may have to learn about the methods of generalisation (i.e. analytic generalisation and transferability, rather than statistical generalisation [41]) to help to interpret legitimately the meaning and significance of the data.

While the Framework Method is amenable to the participation of non-experts in data analysis, it is critical to the successful use of the method that an experienced qualitative researcher leads the project (even if the overall lead for a large mixed methods study is a different person). The qualitative lead would ideally be joined by other researchers with at least some prior training in or experience of qualitative analysis. The responsibilities of the lead qualitative researcher are: to contribute to study design, project timelines and resource planning; to mentor junior qualitative researchers; to train clinical, lay and other (non-qualitative) academics to contribute as appropriate to the analysis process; to facilitate analysis meetings in a way that encourages critical and reflexive engagement with the data and other team members; and finally to lead the write-up of the study.

## Conclusion

We have argued that Framework Method studies can be conducted by multi-disciplinary research teams that include, for example, healthcare professionals, psychologists, sociologists, economists, and lay people/service users. The inclusion of so many different perspectives means that decision-making in the analysis process can be very time consuming and resource-intensive. It may require extensive, reflexive and critical dialogue about how the ideas expressed by interviewees and identified in the transcript are related to pre-existing concepts and theories from each discipline, and to the real ‘problems’ in the health system that the project is addressing. This kind of team effort is, however, an excellent forum for driving forward interdisciplinary collaboration, as well as clinical and lay involvement in research, to ensure that ‘the whole is greater than the sum of the parts’, by enhancing the credibility and relevance of the findings.

The Framework Method is appropriate for thematic analysis of textual data, particularly interview transcripts, where it is important to be able to compare and contrast data by themes across many cases, while also situating each perspective in context by retaining the connection to other aspects of each individual’s account. Experienced qualitative researchers should lead and facilitate all aspects of the analysis, although the Framework Method’s systematic approach makes it suitable for involving all members of a multi-disciplinary team. An open, critical and reflexive approach from all team members is essential for rigorous qualitative analysis.

Acceptance of the complexity of real life health systems and the existence of multiple perspectives on health issues is necessary to produce high quality qualitative research. If done well, qualitative studies can shed explanatory and predictive light on important phenomena, relate constructively to quantitative parts of a larger study, and contribute to the improvement of health services and development of health policy. The Framework Method, when selected and implemented appropriately, can be a suitable tool for achieving these aims through producing credible and relevant findings.

### Summary

•The Framework Method is an excellent tool for supporting thematic (qualitative content) analysis because it provides a systematic model for managing and mapping the data.

•The Framework Method is most suitable for analysis of interview data, where it is desirable to generate themes by making comparisons within and between cases.

•The management of large data sets is facilitated by the Framework Method as its matrix form provides an intuitively structured overview of summarised data.

•The clear, step-by-step process of the Framework Method makes it is suitable for interdisciplinary and collaborative projects.

•The use of the method should be led and facilitated by an experienced qualitative researcher.

## Competing interests

The authors declare that they have no competing interests.

## Authors’ contributions

All authors were involved in the development of the concept of the article and drafting the article. NG wrote the first draft of the article, GH and EC prepared the text and figures related to the illustrative example, SRa did the literature search to identify if there were any similar articles currently available and contributed to drafting of the article, and SRe contributed to drafting of the article and the illustrative example. All authors read and approved the final manuscript.

## Pre-publication history

The pre-publication history for this paper can be accessed here:

http://www.biomedcentral.com/1471-2288/13/117/prepub

## Supplementary Material

Additional file 1 Illustrative Example of the use of the Framework Method. Click here for file
