# Supplementary material for: Using the framework method for the analysis of qualitative data in multi-disciplinary health research
Source: BMC Med Res Methodol. 2013 Sep 18;13:117. doi: 10.1186/1471-2288-13-117 (PMC3848812; doi:10.1186/1471-2288-13-117)
Supplement: Additional file 1 — Illustrative Example of the use of the Framework Method. [file 1471-2288-13-117-S1.docx]

***Additional File 1: Illustrative Example of the use of the Framework Method***

As part of a multi-disciplinary research team including medical sociologists, health psychologists and clinicians, GH, EC & SRe led the analysis of data from a project exploring NHS staff and stakeholders views on providing hospital outpatient paediatric care ‘closer to home’ in community settings.

Heath G, Cameron E, Cummins C, Greenfield S, Pattison H, Kelly D, Redwood S: Paediatric ‘care closer to home’: stake-holder views and barriers to implementation. *Health and Place* 2012, 18(5):1068–1073*].*

In this study, we used semi-structured interviews to collect data from 37 participants including healthcare professionals, managers, commissioners and executive team members. Using the Framework Method, we took a combined approach to analysis, enabling themes to be developed both inductively from the accounts (experiences and views) of research participants and deductively from existing literature. Regular team meetings facilitated our critical exploration of participant responses, discussion of deviant cases and agreement on recurring themes. Examples from this published study will be used throughout this additional file to illustrate each step.

*Section 1: TRANSCRIPTION*

In the ‘Care Closer to Home’ study, to ensure similarity in transcription style across the whole dataset, in the early stages of the project both members of the research team (GH and EC) who were responsible for carrying out transcription examined their transcripts to ensure comparable formatting, until satisfied that any inconsistencies had been resolved. As we were interested in the content, rather than the structure of participants’ responses for analysis, only long pauses, interruptions and nonverbal communication (such as laughter) were noted within the text. We checked all transcripts for errors by listening back to the audio-recording and reading the transcripts simultaneously. We supplemented each transcript with notes made during and immediately after the interview, for example noting background information and instances where views were given after the recorder was switched off.

*Section 2: Familiarisation with the interview*

Members of our research team thoroughly read and re-read each transcript, and listened back to the audio-recorded interviews to become familiar with the whole data set. We found this familiarisation process essential in cases where the researcher analysing the data had not been present during the interview. We also recorded initial impressions in the margins of transcripts, for example where participants expressed exceptionally strong or contrasting views to their colleagues. In our case, this included one GP who, contrary to other GPs, disagreed strongly with the recommendation of moving paediatric outpatient care into the community. Familiarisation through reading and making notes in this way also enabled us to find our way easily around hundreds of pages of transcript later in the analysis.

*Section 3: CODING*

Initially three members of our research team, each from different backgrounds, independently coded the same three transcripts. We underlined interesting segments of text and used the left hand margin to describe the content of each passage with a label or code. This could range from only a few words, to parts of sentences or whole paragraphs. We then used the right hand margin to record more detailed notes and ideas, for example questions to bear in mind as the analysis proceeded, and ideas for explanations or patterns in the data. Below, an excerpt of open coding from one of the researchers is presented. The participant, a GP, talks about how moving care out of the hospital and into more a more comfortable and familiar community setting could facilitate the exchange of information between patients and healthcare professionals. The researcher labelled this as ‘patient experience’ and added notes on how aspects of the place/space of care delivery might influence the medical consultation and overall patient experience. The researcher’s underlining emphasises interesting parts of the data that she felt were worth coding or noting.

*
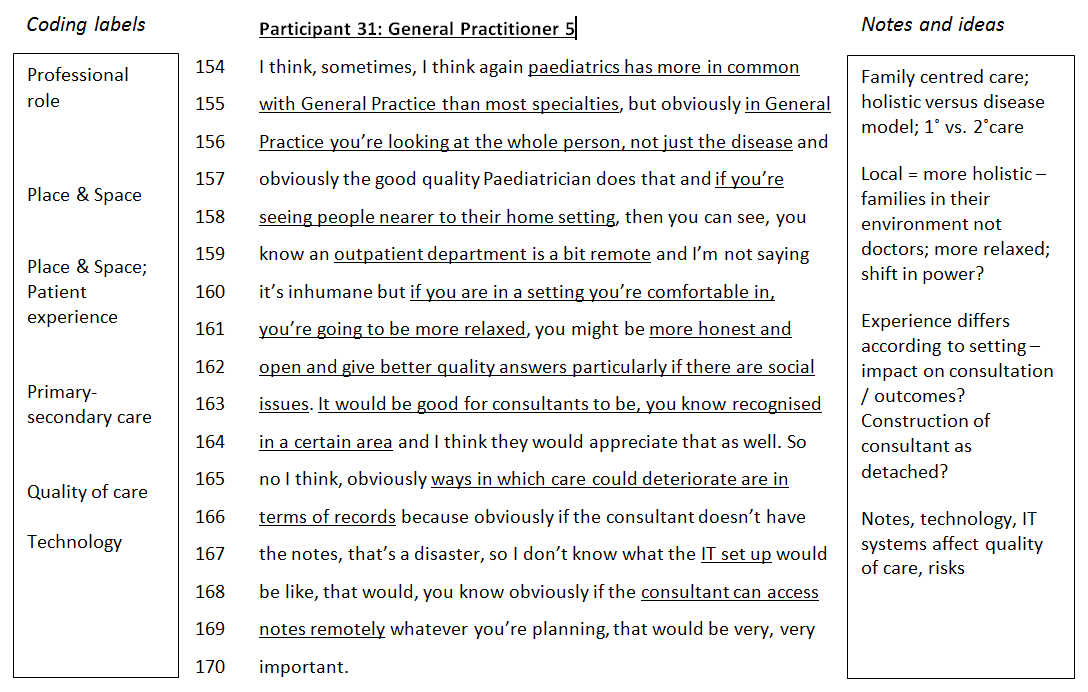
*

*Section 4: Developing a working analytical framework*

After three researchers had each open coded the same three transcripts, we met to discuss the labels we had assigned to each passage. Working through all three, we discussed each coded section in terms of why it had been interpreted as meaningful, what it told us about participants’ views on providing paediatric ‘Care Closer to Home’ and how it might be useful for answering the research question. Generally we highlighted the same passages of text as meaningful. However, sometimes different researchers expressed their interpretations of the content in different ways. For instance, GH coded the first passage of text in the example transcript as ‘professional role’, whereas EC coded it as ‘comparison of specialist Paediatric and General Practice care’. Despite the difference in language, both researchers identified that the participant was discussing similarities and differences between the two specialist roles. We therefore revisited the transcript together and agreed that the term professional role better captured the idea and this code was applied.

After discussion, we agreed on a set of codes, each with a brief definition. This formed the initial analytical framework. Two researchers then independently coded three more transcripts using the initial framework, taking care to note any new codes or impressions which did not fit the existing set. We then met again and following discussion, revised the initial framework to incorporate new and refined codes. At this point we also decided that some codes were conceptually related and therefore should be grouped together. For example, as can be seen in our coding index, four codes (Ideology of ‘closer to home’; Patient-centred approach; Equivalence to Hospital Care; Equity in Service Provision) each related to the underlying principles for guiding the provision of paediatric services. We therefore grouped them together to make an overarching category which we named ‘Philosophy of Care’. The process of refining, applying, and refining the analytical framework was repeated until no new codes were generated. The final framework consisted of sixty-six codes, clustered into thirteen categories, each with a brief explanatory description of their meaning and examples of what ideas or elements might be summarised under that code. Brief explanations of each individual code provided at least some consistency of coding across the team. The example below shows two categories from the final analytical framework with constituent codes, and descriptions of codes.

| **CODE** | **DESCRIPTION** |
| --- | --- |
| **Working Practices** | |
| Professional role | *Perception of own or other’s roles, including empowerment, professional pride, GP commissioning, GPs with Special Interest (GPwSI)* |
| Relationship between primary and secondary care | *Barriers, gaps, advantages and drawbacks, working relationships* |
| Knowledge and skills transfer | *Education, information, explanations, teaching, training (student doctors, GPwSI)* |
| Joined up working | *Instances of working together from two or more different disciplines, working across care sectors* |
| Changes in working practices | *Impact / outcome in terms of changes to working practice (e.g. Saturday clinics), changes to clinician workload, consultant travel* |
| **Philosophy of Care** | |
| Ideology of ‘care closer to home’ (CCTH) | *Attitude towards closer to home agenda and satellite clinic model* |
| Patient-centred approach | *Biopsychosocial & holistic approaches, opportunities for health promotion, patient choice* |
| Equivalence to hospital care | *Comparisons with the hospital, e.g. trying to match standards of care* |
| Equity in service provision | *Distribution of services, equity in access to services, postcode lottery,*  *service distribution* |

*Section 5: Applying the analytical framework*

We applied the final analytical framework to each transcript using the CAQDAS package QSR NVivo version 8. In practice, this meant dividing the transcripts between two researchers and importing them into NVivo ready for indexing. We then systematically went through each transcript, highlighting each meaningful passage of text and selecting and attaching an appropriate code from the final analytical framework. Below is an excerpt from the transcript for ‘Executive 5’ where we highlighted two parts of the text that were relevant for the theme ‘Philosophy of Care’ in which the interviewee discussed access to healthcare services. From the analytical framework, we then selected and attached the code ‘Equity in Service Provision’ and ‘Ideology of Care Closer To Home (CCTH)’. We then used NVivo to share our indexed transcripts, ensuring that each researcher could access the whole data set for the next stage of the process. The example below shows the application of the analytical framework to a transcript, using only codes from the ‘Philosophy of Care’ category.

*
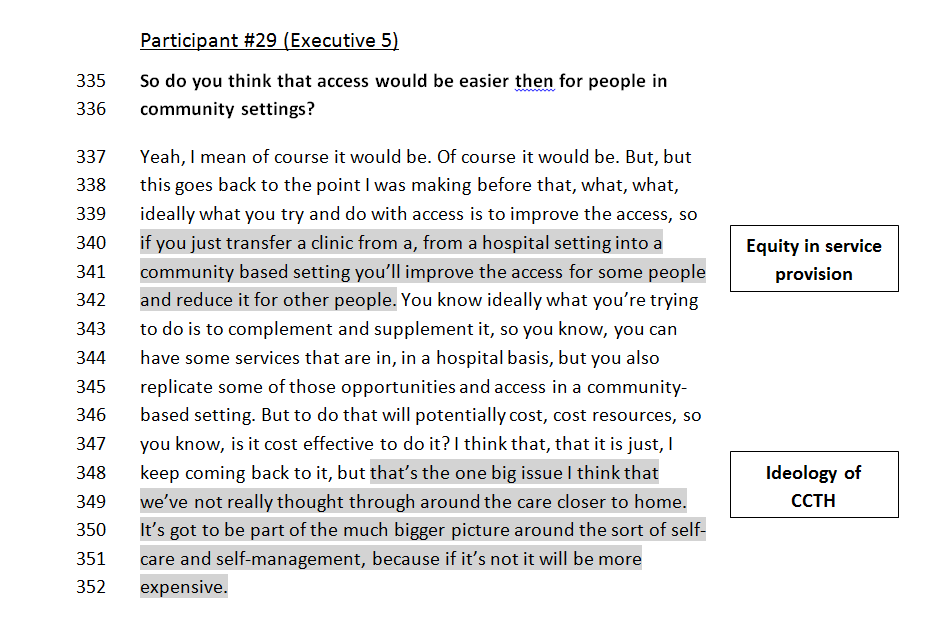
*

*Section 6: Charting data into the Framework matrix*

Once all the data had been coded using the analytical framework, we summarized the data in a matrix for each theme using Microsoft Excel. As illustrated below, the matrix comprised of one row per participant and one column per code. A separate sheet was used for each category. We then abstracted data from transcripts for each participant and code, summarised it using verbatim words and inserted it into the corresponding cell in the matrix. We found NVivo to be invaluable at this stage, as it allowed for quick and easy retrieval of indexed data for specific codes within each transcript. References to potentially interesting quotations were also highlighted within the cells of the matrix using Q or QQ/QQQ depending on how illustrative the quote was. For example, a quote from ‘Manager 1’ clearly demonstrated the point that new paediatric services should respond to the needs of families, rather than the needs of the organisation: ‘we need to be very different in how we deliver services based around what the patients and their families need and I think at the moment we’re not. We’re still focused on what’s easier for us’. This quotation was later used in the published paper. The example below is an extract from the ‘Philosophy of Care’ matrix, with page and line references. Underlining indicates verbatim text. Certain abbreviations were agreed by the team in advance (CCTH: care closer to home, CTH: closer to home, Gen Paeds: General Paediatrics)

|  | **Ideology of CCTH** | **Patient-centred approach** | **Equity in service provision** | **Equivalence to hospital care** |
| --- | --- | --- | --- | --- |
| **Manager 1** | Gen Paeds doesn’t need to be in hospital; with right infrastructure CCTH makes sense *[p1, 24].* | Need to deliver services based on what families need; at the moment focused on what's easier for us **QQQ** *[p16, 467].* | For some people a city centre hospital is CTH than a clinic in the community *[p620, 21].* | Need to instil confidence that they're getting same level of care, but CTH *[p2, 33].* |
| **Consultant 7** | CCTH is a good recommendation; only patients who need specific investigations should attend hospital for outpatients *[p1, 7].* | Preservation of the institution (hospital), rather than needs of the population they actually serve, seems to be the predominant interest **QQ** *[p3, 69].* |  | Make it clear to patients it’s exactly same service in satellite clinic, they are seeing me (same consultant) *[page 1, 16].* |
| **Executive 5** | The more we keep patients out of hospital, the better; don’t want patients in hospital if don’t need to *[p1, 14].* CCTH is part of bigger picture around self care and self management [*p11, 350*]. |  | If just transfer clinic from hospital to community setting, improve access for some, but reduce it for others **QQ** [*p11, 340*]. |  |

*Section 7: INTERPRETING THE DATA*

Themes were generated from the data set by reviewing the matrix and making connections within and between participant and categories. This process was influenced both by the original research objectives and by new concepts generated inductively from the data. During the interpretation stage, we tried to go beyond descriptions of individual cases towards developing themes which offered possible explanations for what was happening within the data. Ideas were generated, explored and fleshed out through the use of analytical memos and discussion within the team. Below is an example of a memo that was written about the category ‘Philosophy of Care’ to develop the idea of tensions between the ideology of providing care closer to home and the practicalities of delivering it on the ground. We structured the memo with sub-headings, including a definition of the category, specific codes that related to it, a summary of the raw data, discussion of any deviant cases, and further points for consideration and comparison. We also used bullet points, bold and italic fonts and underlining to look for patterns within the data and also included illustrative quotations in bold with references to the original transcripts. This memo was later incorporated into the final theme “Care closer to home: a moral imperative impossible to deliver?” in the published paper.

**MEMO: ‘Philosophy of Care’**

Definition

**Ideology versus practicality**: Care Closer To Home (CCTH) is perceived as desirable but difficult to achieve. Financial and practical difficulties (e.g. ensuring equivalence in standards and equity in service provision) challenge the philosophical ideology underpinning paediatric CCTH.

Codes

Ideology of CCTH; Patient-centred approach; Equivalence to hospital care; Equity in Service Provision

Summary of data

- Ideology and patient-centeredness

Participants view paediatric CCTH as intrinsically desirable, a sound theoretical principle for keeping children out of hospital and guiding health service redesign: ***“Only the patients who need specific investigations they can only get in the hospital really need to attend the hospital.”* (Consultant 7, p. 1, line 7)**. In addition, participants were keen to convey their support for a user-led agenda in which new services incorporate families’ perspectives, as well as being responsive to the needs of communities. This was contrasted with the present service design which was perceived as reflecting the needs of the organisation: ***“We need to be very different in how we deliver services based around what the patients and their families need and I think at the moment we’re not, we’re still focused on what’s easier for us”* (Manager 1, p. 20, line 464);**

***“The preservation of the institution, rather than the needs of the population they actually serve, seems to me to be the predominant interest”* (Consultant 7, p. 3, line 69).**

The ideology of providing care that is both closer to patients’ homes and tailored to their needs was further contrasted with the practical and financial difficulties of delivering ‘hospital’ services in community settings. So, although closer to home policies were philosophically presented as unproblematic, the process of actually setting up and maintaining clinics in terms of finance and infrastructure was seen as far more challenging: “*What we’re talking about is logistics and possibilities and that’s not necessarily the same as kind of philosophical approach is it?”* (Consultant 8, p. 14, line 324).

Does this mean that participants supported CCTH in theory, but not in practice? *“Behind it in theory but the practice is often more complex than the theory”* (Executive 1, p. 4, line 73). Does this call into question the interviewee’s commitment to delivering paediatric CCTH? Perhaps the reluctance of participants to fully commit to implementing this policy relates to uncertainty about whether a new government will endorse the initiative? Given that interviews spanned the introduction of a new government / White Paper, the political and economic context was clearly present in participants’ views, leading many to examine the costs and benefits of CCTH: ***“We’ve got to strike the balance between improving access and improving choice... And what’s actually affordable”* (Executive 5, p. 16, line 364)**.

- Equity, equivalence and fairness

The CCTH policy was also contested on ethical grounds. Many participants depicted a moral obligation of providing outpatient services in community settings which were of at least an equivalent standard to hospital care: *“You would never want to take something out into the community that’s any different than you’d be happy providing here”* (Manager 3, p. 10, line 226). Participants also suggested that if patients were given appointments on the basis of their geographical location, this could create a ‘postcode lottery’ in which access to paediatric health services is defined by the area in which a patient lives: *“If you start pulling patients out of, based on their geographical area from the total waiting list... people in that particular clinic might be seen earlier if it’s a first appointment... So that kind of might create a double standard”* (Consultant 1, p. 7, line 205). Thus, far from having the desired outcome of improving access, some participants suggested that decentralisation of services may actually reduce access for some families: ***“If you just transfer a clinic from a hospital to a community setting, you’ll improve access for some and reduce it for others”* (Executive 5, p. 11, line 340)**.

Deviant cases

One G.P did not think that paediatric outpatient care should be moved closer to patients’ homes. However, the GP’s surgery is geographically located close to the hospital, which could explain his views?

Points for further consideration

- What are participants’ motivations for putting policy into practice?
- What is the CCTH agenda intended to achieve (e.g. keeping children out of hospital, improving access, relieving demand on hospitals, reducing DNA)?
- Are consumerist ideals (e.g. convenience and satisfaction) compatible with sustainability in the NHS?
